# Neurobiological pathways to Alzheimer’s disease: Amyloid-beta, TAU protein or both?

**DOI:** 10.1590/S1980-57642009DN30300003

**Published:** 2009

**Authors:** Vanessa de Jesus R. de Paula, Fabiana Meira Guimarães, Breno Satler Diniz, Orestes Vicente Forlenza

**Affiliations:** 1 Laboratory of Neuroscience LIM-27, Department and Institute of Psychiatry, Faculty of Medicine, University of São Paulo, SP, Brazil.

**Keywords:** Tau protein, amyloid precursor protein, beta amyloid, Alzheimer’s disease

## Abstract

Alzheimer’s disease (AD) is a neurodegenerative disease characterized by progressive cognitive decline, including memory loss, behavioral and psychological symptoms and personality changes. The neuropathological hallmarks of AD are the presence of neuritic (senile) plaques (NP) and neurofibrillary tangles (NFT), along with neuronal loss, dystrophic neurites, and gliosis. Neuritic plaques are extracellular lesions and their main constituent is the amyloid-β_42_ peptide (Aβ_42_). Neurofibrillary tangles are intracellular lesions that are mainly composed of hyperphosphorylated Tau protein. In this article, we review the major hypotheses concerning the physiopathology of AD, focusing on the β-amyloid cascade as primary events (supported by the “βaptists”) and cytoskeletal abnormalities secondary to the hyperphosphorylation of protein Tau (as advocated by the “Tauists”). We further provide an integrative view of the physiopathology of AD.

## Abstract (pt)

A doença de Alzheimer (DA) é uma desordem neurodegenerativa progressiva que cursa comprometimento da memória e outras funções cognitivas, alterações comportamentais, psíquicas e da personalidade. Os achados neuropatológicos característicos da DA são as placas neuríticas (senis) e os emaranhados neurofibrilares, também ocorrendo distrofia de neuritos, gliose e perda neuronal. As placas neuríticas são lesões extracelulares que têm no peptídeo beta-amilóide (Aβ_42_) seu principal constituinte. Os emaranhados neurofibrilares são lesões intraneuronais compostas por agregados de proteína TAU em estado hiperfosforilado. Neste artigo de revisão, apresentamos as principais hipóteses relacionadas à fisiopatologia da DA, com foco na cascata do amilóide como evento inicial (hipótese preconizada pelos “βaptistas”) e nas alterações do citoesqueleto neuronal, decorrentes da fosforilação anormal da TAU (conforme proposto pelos “tauístas”). Os achados são discutidos numa leitura integrada desses dois mecanismos fisiopatológicos.

Alzheimer’s disease (AD) is a progressive neurodegenerative disease that accounts for the majority (50 to 75%) of dementia cases in clinical and population samples.^1,2^ The clinical manifestations of AD include the progressive deterioration of episodic memory and other intellectual abilities, leading to global cognitive decline, behavioral and psychiatric symptoms and personality changes. Most AD cases begin after 65 years old (i.e. late-onset AD). However, some cases manifest in younger subjects (i.e. early-onset AD). The dementia syndrome in these patients usually begins in the 5^th^ or 6^th^ decade of life.

Age and low-educational status are the most important risk factors for late-onset AD.^3^ Other important risk factors include the presence of the allele ε4 of the apolipoprotein E (APOE) gene, history of head trauma with loss of consciousness, life-long uncontrolled cardiovascular risk factors (hypertension, diabetes mellitus, dyslipidemia), sedentary life style, life-long low cognitive demand.^4^ On the other hand, early-onset AD is usually associated to genetic mutations, the most commonly described being the Amyloid Precursor Protein (APP) gene on chromosome 21, and the Presenilin 1 and 2 genes on chromosomes 14 and 1, respectively.^5^

Regardless of age of dementia onset, the neuropathological findings in AD patients are very similar. The hallmarks of AD are the presence of senile (neuritic) plaques (NP) and neurofibrillary tangles (NFT), together with neuronal loss, dystrophic neurites, and gliosis.^6^ Neuritic plaques are extracellular lesions and their main constituent is the amyloid-β_42_ peptide (Aβ_42_). Neurofibrillary tangles are intracellular lesions and are mostly composed of hyperphosphorylated TAU protein. Despite controversial findings, the progression of the clinical AD dementia syndrome correlates with the pattern of progression of these lesions in the brain.^7^ In this study, we review the mechanisms involved in the amyloidogenic shift of APP metabolism, along with the cytoskeletal changes that arise from abnormal regulation of TAU phosphorylation. We thus address two major hypotheses in the pathophysiology of AD, namely the β-amyloid cascade (the “βaptists”) and the hyperphosphorylation of TAU (the “Tauists”).

In order to address the current state of knowledge regarding the role of TAU and amyloid cascade in the pathogenesis of AD, we performed a comprehensive, open review of the literature. We additionally used the key-words “TAU”, “amyloid precursor protein”, “(beta)-amyloid”, “Abeta”, “GSK”, “Alzheimer’s disease” to search the Pubmed and Scielo databases for articles published between 2000 and 2009. Secondary references were also obtained from the reference list of relevant papers, or based on seminal contributions in this field.

## The amyloid precursor protein/amyloid-β metabolism and function

**Figure 1:**
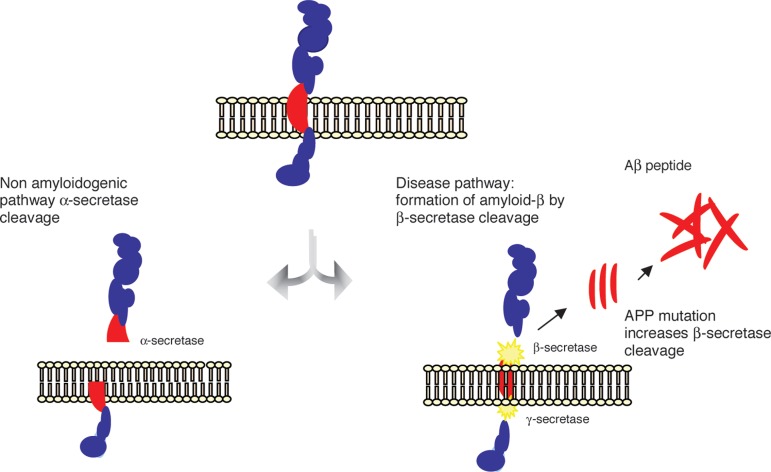
The amyloid precursor protein (APP) is a transmembrane protein cleaved by secretase enzymes. In the non-amyloidogenic pathway, APP is cleaved preferentially by a-secretase. In the amyloidogenic pathway, neurotoxic Ab peptides are released after sequential cleavage of APP by b and g-secretases, and further accumulate into oligomeric aggregates.

APP is a transmembrane protein and represents one of the most abundant proteins in the central nervous system (CNS). It is also expressed in peripheral tissues, such as epithelium and blood cells.^8^ APP is alternatively metabolized by two distinct routes, i.e., the non-amyloidogenic and the amyloidogenic pathways. In the former, APP is cleaved by the enzyme γ-secretase, releasing a soluble N-terminal fragment (sAPPα) and a C-terminal fragment (C83), which is further cleaved by α-secretase, releasing a C-terminal fragment of 3KDa (C3). The cleavage of APP by α-secretase occurs within the region that contains the Aβ peptide, precluding the formation of this peptide.^9^ Alternatively, APP may be cleaved by β-secretase releasing a smaller N-terminal fragment (sAPPβ) and a C-terminal fragment (C99) that produces the full-length β-amyloid peptides (Aβ) upon the subsequent cleavage by γ-secretase (Figure 1).

There are several Aβ peptide species, where those with 40 and 42 amino acids (Aβ_40_ and Aβ_42_) are the most abundant in the brain.^10^ Aβ species are released as monomers which progressively aggregate into dimers, trimers, oligomers, protofibrils and fibrils, to finally deposit and originate diffuse, and then, mature amyloid plaques^9^. Despite their similarities, Aβ_42_ is more prone to aggregation and fibrilization, being the most toxic Aβ peptide and playing a pivotal role in the pathogenesis of AD.^11^

The Aβ oligomers are considered the most toxic form of the Aβ peptide.^12^ They interact with neurons and glial cells leading to the activation of inflammatory cascades, oxidative stress,^13^ deregulation of calcium metabolism and TAU phosphorylation, and induction of neuronal apoptosis.^10^ Together, these phenomena give rise to a self-perpetuating, positive feedback loop in which the production of Aβ peptides leads to deleterious events to the neuronal cells, which in turn contributes to dysfunctional APP metabolism and further production of Aβ peptides. amyloid-β fibrils deposit in neuritic plaques in a sequential pattern: diffuse neuritic plaques, mature neuritic plaques, senile plaques and phantoms of senile plaques in more advanced stages of the disease. The plaque formation also has a deleterious impact on neuronal homeostasis, also leading to dysfunction and, ultimately, neuronal death.^14^

Under physiological conditions, the APP is preferentially metabolized by the non-amyloidogenic pathway and there is equilibrium between the production of Aβ peptides and their clearance from the brain.^15^ Currently, two proteins are deemed as heavily involved in the clearance of Aβ peptides from the brain: apolipoprotein E (APOE) and the insulin degrading enzyme (IDE). The exact mechanism (or mechanisms) by which Aβ peptides are cleared from the brain has not been established, but a current hypothesis holds that these proteins bind Aβ peptides, inhibiting their aggregation into oligomers and thus facilitating their clearance from brain tissue.^13^ Under pathological conditions, there is a metabolic shift favoring the amyloidogenic cleavage of APP which, along with a reduction of Aβ clearance, leads to the accumulation of Aβ within the brain.^16^

## TAU protein metabolism and function

**Figure 2:**
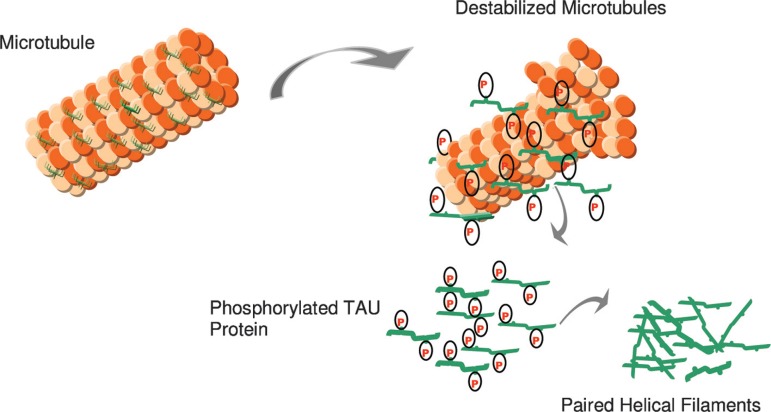
In AD, there is a reduction in the ability to bind microtubules and promote microtubule assembly. Hyperphosphorylated Tau may contribute to a destabilized microtubule network, impaired axonal transport, and ultimately in neurofibrillary tangle (NFT) formation and neuronal death.

The TAU protein is a microtubule-associated protein found in most tissues and which is highly expressed in the central and peripheral nervous system. It is an important component of the neuronal cytoskeleton,^17^ given its ability to interact with α- and β-globulin to stabilize the microtubules^18^ (Figure 2). In neurons, the microtubules are essential for the maintenance of neuronal structure, axonal transport, and synaptic plasticity.^19^

Six TAU isoforms are described in mammals. The main differences between these isoforms hinge on the existence of three or four binding domains to tubulin, in addition to some minor differences in the N-terminal end of the protein.^20-23^ The interaction between TAU and tubulin is a dynamic process in which TAU promotes its own polymerization and inhibits fast depolymerization of the tubulin.^24^ This process is regulated by the phosphorylation state of TAU protein, which comprises approximately 79 phosphorylation sites at serine and threonine residues.^25^ The balance between phosphorylation and dephosphorylation of these epitopes promotes conformational changes that influence how TAU protein interacts with α- and β-tubulin and that stabilize the microtubules in the neurons.^26^ Several protein kinases and phosphatases are involved in the regulation of TAU phosphorylation, the enzyme glycogen synthase kinase 3β (GSK3β) being the most important TAU-kinase in the neurons.^22^

A reduction of the expression of certain phosphatases was also identified in cerebral tissues of patients with AD, namely PP1, PP2A and PP5.^27^ The majority of the residues of serine and treonine that are found in a hyperphosphorylated state in fetal TAU and in PHFs is followed by proline, suggesting that TAU kinases belong to the family of proline-directed enzymes,^28^ including cyclin-dependent kinases (CDC2, CDK5, TPKll), some enzymes of the family of the mithogen-activated kinases (MAPK), and GSK3β.^29^ Such enzymes are capable of phosphorylating Tau in vitro and have been detected in the cellular strata of cerebral tissue of individuals with AD. Other non-proline directed kinases have also been identified in neurofibrillary tangles and are important for the regulation of TAU phosphorylation, modulating the activity of the former enzymes. PKC, casein-kinases l and ll,^30^ calcium calmodulin-kinase ll (CaMPK-ll),^31^ and protein kinase A (PKA) are examples of this group of enzymes.

At embryonic stages, neuronal TAU is predominantly found in a hyperphosphorylated state. This is due to the great demand for neuroplastic changes in neurons and synapses at early developmental stages of the central nervous system.^31^ On the other hand, in the adult, mature central nervous system, dephosphorylated TAU is required for microtubule stability, necessary for the maintenance of cytoskeletal homeostasis, both at structural and functional levels.^22^ Dynamic changes in the phosphorylation state of TAU occur in mechanisms involved in neuronal remodeling and synaptic plasticity.^32^ Under pathological conditions, including the pathophysiology of AD, TAU can be abnormally hyperphosphorylated, which impairs its capacity to bind to tubulin, leading to destabilization of the microtubule structure^33^ and impairment of axonal transport and synaptic metabolism. Such changes eventually result in the collapse of the cytoskeleton, loss of cellular viability and neuronal death.^34^

## The amyloid-β cascade hypothesis: the “βaptist doctrine”

The amyloid-β cascade hypothesis was first described in the early 1990s^35^ and a comprehensive review was recently published by Korczyn (2008).^36^ It states that the accumulation of β-amyloid peptides into neuritic and senile plaques in the brain, due either to their increased production or decreased clearance, is the core pathogenetic feature of AD. The accumulation of Aβ triggers several neurotoxic events, such as mitochondrial dysfunction, increased oxidative stress, abnormal neuroinflammatory response,^13^ decreased neurotrophic support, decreased neuroplasticity and neurogenesis, hyperphosphorylation of TAU, apoptosis and disruption of calcium homeostasis. These events occur in a positive feedback loop, amplifying Aβ neurotoxicity and culminating in neuronal death.^13^ It is not only the accumulation of A? which triggers these hazardous events, as the oligomeric and fibrillar forms of the Aβ peptide also interact with neurons and glial cells triggering a series of negative events. In fact, the non-aggregated Aβ forms are currently regarded as the most toxic forms of this protein.

The amyloid-β cascade hypothesis was based mostly on *in vitro* and *in vivo* studies and was further strengthened by the identification of genetic mutations associated with early-onset AD (e.g. mutation in the APP gene, and presenilin 1 and 2 genes), and the development of genetically modified animal models expressing mutant DNA.^5^ These mutations lead to a massive overproduction of Aβ and subsequent aggregation into oligomers, and deposition in plaques.

There are several open issues regarding the amyloid-β cascade hypothesis. First, neuropathological studies found no significant correlation between amyloid plaque density in the brain and severity of dementia. On the other hand, extensive neuronal and synaptic losses are found in the earliest stages of the disease and are better correlated with the cognitive deficits and severity of dementia in these patients.^6,36-38^ AD is the only neurodegenerative disease in which the Aβ peptide is allegedly the pathological keystone; in contrast, post-mortem studies of the ageing brain show that non-demented individuals may have amyloid plaques similar to those found in AD patients.^39^ Also, most anti-amyloid based therapeutic strategies have failed to show clinically relevant results either in improving cognition or in halting the clinical progression of dementia.^40,41^ Finally, cellular and animal models of AD are based largely on genetic mutations associated with familial, early-onset AD (EOAD), which accounts for a small proportion of dementia cases. Since late-onset AD (LOAD) represents the vast majority of cases, and considering that amyloidogenesis in these patients occurs to a lesser extent compared to the early AD, questions have been raised concerning the appropriateness of EOAD models to aid understanding of LOAD.^42^

## TAU protein and Alzheimer’s disease (“the Tauists)”

The intra-neuronal lesions called neurofibrillary tangles (NFT)^43^ were in fact those described by Alois Alzheimer in the beginning of the nineteenth century. The main component of NFTs are the paired helical filaments (PHFs) of hyperphosphorylated TAU (for a review, see Mandelkow et al., 2007).^46^ At least 25 abnormal phosphorylation sites have been described in AD,^41^ constituting a marker of neuronal degeneration in this disorder.^45^ The abnormal phosphorylation of TAU in serine/threonine residues near the tubulin binding domain favors disaggregation of the TAU-tubulin complex and the reassembly of TAU into PHFs, giving rise to NFT formation.^33^ Due to the importance of TAU in the maintenance of neuronal stability and homeostasis, its hyperphosphorylation leads to a cascade of events that ultimately cause neuronal death.

There are several lines of evidence that support the notion that the dysfunction in TAU homeostasis is a primary event in AD, a phenomenon also found in other neurodegenerative disorders, such as frontotemporal dementia (Pick’s disease) and multiple system atrophy.^33^ Neuropathological studies have shown that the distribution of NFTs in the brain correlates with the clinical progression of dementia in AD.^7^ Moreover, intra-neuronal hyperphosphorylated TAU can be found in brain of subjects with very mild dementia, unaccompanied by β-amyloid pathology.^45,47,48^ Therefore, TAU hyperphosphorylation may be an early event in the physiopathology of AD, whereas other pathological mechanisms, including Aβ overproduction and activation of inflammatory cascades and oxidative stress, may be secondary to the overall dysfunction in neuronal homeostasis.^39^ However, the larger body of evidence linking amyloid-β to AD, along with the lack of genetic mutations in the TAU gene associated to early or late-onset AD, weaken the hypothesis that abnormalities in TAU may be the trigger of other pathologic events in AD.^13^

## The GSK3β hypothesis

Despite the strong evidence supporting the primary role of Aβ peptides and hyperphosphorylated TAU in the pathogenesis of AD, neither hypothesis satisfactorily accounts for the full spectrum of pathological processes associated with this disease. In addition, alternative hypotheses suggest that NFTs and Aβ plaques may be independent epiphenomena in the course of AD.^6,38^ Therefore, alternative mechanisms have been proposed, most of which involve the activity of upstream enzymes that play a role in these two cascades. GSK3β is a key enzyme in the regulation of cellular metabolism including TAU phosphorylation. Wnt signaling may represent another important aspect in the neurobiology of AD, since Wnt signaling leads to the inactivation of GSK, preventing TAU phosphorylation in the GSK-dependent epitopes.^49,50^ In AD, GSK3β has been found in a hyperactive state, leading to hyperphosphorylation of TAU. Recent studies have also demonstrated that GSK3β also regulates APP metabolism, and is conducive to amyloidogenic cleavage (and thus the overproduction of Aβ), reduced neurogenesis and increased apoptosis.^46^

*In vitro* studies have shown that the pharmacological activation of GSK3β leads to neuronal changes and death in a similar fashion to that observed in AD.^50,53^ On the other hand, *in vitro* and *in vivo* studies have demonstrated that the pharmacological inhibition of GSK3β activity (e.g. with lithium salts) protects against neuronal degeneration and death induced by A? and Tau hyperphosphorylation.^50-54^ Few studies have been carried out in humans to determine the activity of GSK3β in AD patients. One interesting study in human leukocytes showed that peripheral GSK3β activity is increased in AD.^55^ Such converging evidence support the “GSK3β hypothesis” in AD,^56^ according to which, the deregulation of GSK3β metabolism leading to increased activity is an early pathological event in the pathophysiology of AD, triggering several downstream events culminating in increased production of Aβ and TAU hyperphosphorylation. Despite the elegant mechanisms elicited by the GSK3β hypothesis, which encompasses in a broader sense both “baptists” and “Tauists”, it lacks consistent empirical evidence to go beyond the prevailing hypotheses.

## Conclusions

Alzheimer’s disease is a complex neurodegenerative disease with a multi-factorial etiology. Current hypotheses, namely the amyloid cascade and TAU pathology, only partially explain the pathophysiology of the disease. It is likely that no single hypothesis will be able to account for all the aspects that underlie the disease process, or to differentiate pathological from normal brain ageing. Given the evidence reviewed in the present study, amyloid deposition and production and/or tau hyperphosphorylation in the brain could be an epiphenomena in the course of AD and not the mainstream features of its pathogenesis.^36,37^ New concepts such as brain and cognitive reserve, neuroplasticity, neurogenesis, restorative functions and neuronal resilience to chronic insults, should be explored to better understand the physiopathology of this complex disorder.
